# Relationship between interstitial glucose variability in ambulatory glucose profile and standardized continuous glucose monitoring metrics; a pilot study

**DOI:** 10.1186/s13098-020-00577-5

**Published:** 2020-08-12

**Authors:** Akemi Tokutsu, Yosuke Okada, Keiichi Torimoto, Yoshiya Tanaka

**Affiliations:** First Department of Internal Medicine, School of Medicine, University of Occupational and Environmental Health, 1-1 Iseigaoka, Yahatanishi-ku, Kitakyushu-Shi, 807-8555 Japan

**Keywords:** Ambulatory Glucose Profile (AGP), Flash Glucose Monitoring (FGM), Inter-quartile Range (IQR), Retrospective study

## Abstract

**Background:**

Treatment indexes using continuous glucose monitoring (CGM) have become standardized internationally, and the use of ambulatory glucose profile (AGP) is currently recommended. However, the relationship between AGP indexes and standardized CGM metrics has not been investigated. Using flash glucose monitoring (FGM), this retrospective study served to evaluate the association of the inter-quartile range (IQR) of AGP with standardized CGM metrics.

**Methods:**

The study subjects were 30 patients with type 2 diabetes mellitus (T2DM) and 23 non-diabetic patients (control group). We evaluated average IQR (AIQR) and standardized CGM metrics. The primary endpoint was the relationship between AIQR and Time in range (TIR) in a 24-h period.

**Results:**

In the T2DM group, the AIQR was notably high and correlated negatively with TIR, and positively with Time above range, average interstitial glucose level, standard deviation of interstitial glucose, coefficient of variation of interstitial glucose, and mean of daily difference in blood glucose (MODD). For the T2DM group, the AIQR was notably lower in patients who achieved TIR > 70%, compared to those who did not. The AIQR cutoff value, as determined by ROC analysis, was 28.3 mg/dl for those who achieved TIR > 70%. No association was detected between the presence of hypoglycemia and AIQR.

**Conclusions:**

Our study is the first to provide the AIQR cutoff value for achieving the TIR target value. The range of interstitial glucose variability in AGP was associated with indexes of intra- and interday variations and hyperglycemia. Our results provide new perspectives in the yet-to-be established methods for evaluation of AGP in practical clinical settings.

## Background

Continuous glucose monitoring (CGM) and flash glucose monitoring (FGM) are becoming standard devices used for management of glucose levels in diabetic patients. A consensus report on CGM metrics was published for the first time last year, and the time-in-range (TIR) and blood glucose levels of ≥ 180 and < 70 mg/dl have become international standards [1] as diabetes treatment indexes. A study using DCCT research data indicated that TIR correlates with retinopathy and nephropathy [2], while the percentage of low blood glucose under 70 is associated with the risk of serious hypoglycemia [3].

On the other hand, the use of the ambulatory glucose profile (AGP) as a standard CGM report has been recommended [1]. The AGP proposed by Mazze et al. [4] is an analytical method that allows easy and visual understanding of various factors, such as the time during the day when there is a high probability of development of hypoglycemia or hyperglycemia, and times during the day with large swings in interstitial glucose levels; the AGP can also indicate movements and trends in interstitial glucose variability (Additional file 1: Figure S1). Although indexes, such as the inter-quartile range (IQR), inter-decile range (IDR) and median are the leading AGP indicators, their relationships with the existing standardized CGM metrics have not been identified; furthermore, the target values and cutoff values in T2DM have not been proposed.

In this pilot study, we used FGM and evaluated the inter-quartile range (IQR) of AGP and its association with standardized CGM metrics. We also examined the target values and cutoff values in T2DM.

## Materials and methods

### Subjects

We conducted a retrospective study that included 30 patients with type 2 diabetes mellitus (T2DM) and 23 non-diabetic patients who attended the outpatient clinic of the University of Occupational Medicine Hospital and University of Occupational Medicine Wakamatsu Hospital, between September 2018 to January 2019. At the time of the study, the T2DM patients were being treated for T2DM and monitored with the flash glucose monitoring system (FGMS^®^ System FreeStyle Libre Pro System, Abbott Diabetes Care, Inc.) for at least 8 days. The following inclusion criteria were applied in the selection of the T2DM group: (1) Patients aged between 30 and 80 years at the time of consent to the study; (2) Outpatients with T2DM, who were examined clinically by diabetologists and tested for GAD antibodies as well as other tests to exclude type 1 diabetes and latent adult-onset autoimmune diabetes (LADA); (3) Patients who had not changed (added, switched, or discontinued) their glucose-lowering medications or changed the doses of these medications within 4 weeks before the start date of monitoring with the FreeStyle Libre Pro sensor. We also applied the following exclusion criteria: (1) Type 1 or secondary diabetes mellitus; (2) Patients with severe infections or serious trauma, and pre- and postoperative patients; (3) Patients on dialysis; (4) Patients with severe liver dysfunction (AST 100 IU/l or greater); (5) Patients with moderate or serious heart failure (NYHA/New York Heart Association Classification III or higher stage); (6) Pregnant, lactating, or planning to become pregnant patients.

Patients and hospital staff confirmed to be non-diabetic and had no glucose intolerance were recruited as the non-diabetic control group.

The study protocol and opt-out method of informed consent were approved by the ethics committee of the University of Occupational and Environmental Health (Trial registration: H27-186, Registered 25 Dec 2015).

We used the following baseline definition of diabetic microangiopathy: The earliest clinical evidence of nephropathy is the appearance of low but abnormal levels (≥ 30 mg/day or 20 μg/min) of albumin in the urine, referred to as microalbuminuria [5]. The urinary albumin excretion rate is presented as the albumin-to-creatinine ratio (mg/g creatinine).

In this study, the albumin-to-creatinine ratio was measured in single voided urine samples and microalbuminuria was defined as 30–300 mg/g creatinine. Diabetic retinopathy was defined as simple or more severe retinopathy based on funduscopic examination by ophthalmologists. Diabetic neuropathy was diagnosed by the presence of two or more clinical symptoms (e.g., bilateral spontaneous pain, hypoesthesia, paresthesia of the legs), absence of ankle tendon reflexes, and decreased vibration sensations using a C128 tuning fork.

### Flash glucose monitoring system

The device automatically measures glucose concentration in the interstitials every 15 min and records the interstitial glucose level 96 times a day. The calculation can be performed using the instructions provided by Miscrosoft^®^ (Redmond, WA) Excel and the EasyGV (available free for non-commercial use at www.easygv.co.uk) The average interstitial glucose level (AG), median, standard deviation (SD), coefficient of variation (CV), percent time at interstitial glucose level of 70–180 mg/dl (TIR; time-in-range), percent time with interstitial glucose level at > 180 mg/dl (TAR; time-above-range), percent time with interstitial glucose level at < 70 mg/dl (TBR; time-below-range), maximum, minimum, glucose management indicator (GMI, new terms used for estimating HbA1c from CGM) [6], mean of daily difference in blood glucose (MODD), low blood glucose index (LBGI), high blood glucose index (HBGI), and AGP 25–75th percentile width (IQR; interquartile range) were obtained from the data recorded with the FGM [7, 8]. Hypoglycemia was defined as interstitial glucose value < 70 mg/dl, as measured by FGM. The FGM recorded and stored interstitial glucose levels over seven consecutive days. Data collected over the first day were discarded to avoid bias due to the insertion and removal of FGM, or insufficient stability of the monitoring system. We recorded the daily average value, and listed the average value of 7 days.

### Laboratory tests

HbA1c (%) was measured by HPLC using Tosoh HLC-723 G8 (Tosoh Co., Kyoto, Japan), and recorded as a NGSP (National Glycohemoglobin Standardization Program) value. eGFR (estimated glomerular filtration rate) was calculated as 194 × serum creatinine concentration (mg/dl) − 1.094 × age − 0.287 in men, and 194 × serum creatinine concentration (mg/dl) − 1.094 × age − 0.287 × 0.739 in women.

### Statistical analysis

Data are shown as mean ± standard deviation. The Shapiro–Wilk normality test was used to check the distribution of data. Differences between the mean values of various parameters in two groups were tested for statistical significance using the Student’s *t* test after confirming equal variance by the F test and Welch’s t-test, or the presence of normal distribution. The Mann–Whitney U test was used for data with skewed distribution, and Spearman’s correlation analysis was used for relationships between two variables. We also analyzed the cutoff values using the Receiver Operating Characteristic (ROC) curve. The required sample size in ROC analysis was calculated as 25 patients in total using an area under the curve (AUC) of 0.85, test power of 0.80, significance level of 5%, and a 5:1 ratio for the group that achieved TIR > 70% versus the group that did not achieve TIR > 70%. A *p* value < 0.05 denoted the presence of statistical significance. All statistical procedures were performed using the SPSS Statistical software version 25.0 (SPSS Inc., Chicago, IL).

## Results

### Clinical characteristics of study participants

None of the patients reported sensor-related problems, such as skin peeling and skin irritation, to warrant dropout/withdrawal from the study. Table 1 lists the clinical characteristics of the study subjects. They comprised 53 subjects (22 males, 31 females). Of these, 23 formed the non-diabetic control group (5 males and 18 females), with a mean age of 43.5 ± 16.5 years (range, 19–88) and BMI of 22.0 ± 3.3 kg/m^2^. The diabetic group consisted of 30 patients (17 males, 13 females) with a mean age of 68.5 ± 7.4 years (range, 51–79) and BMI of 24.1 ± 3.9 kg/m^2^, HbA1c of 6.7 ± 0.6% (range, 5.7–7.7), and disease duration of 14.7 ± 11.5 years (range, 1.3–43.0). For the diabetic group, 70% of the patients were using DPP-4 inhibitors and 50% were on biguanides. Among the study group, 6 were insulin users, with 3 using the long-acting basal insulin only, 1 using a mixture formulation of long-acting basal insulin and rapid-acting bolus insulin, and 2 using intensive insulin therapy.

**Table 1:** Baseline characteristics of the 53 patients

	Mean ± SD	
	Control group (n = 23)	Diabetic group (n = 30)
Sex (male/female)	5/18	17/13
Age (year)	43.5 ± 16.5	68.5 ± 7.4
Height (cm)	165.2 ± 11.3	160.7 ± 8.4
Weight (kg)	60.9 ± 15.0	62.6 ± 13.4
BMI (kg/m^2^)	22.0 ± 3.3	24.1 ± 3.9
SBP (mmHg)	116.2 ± 11.5	134.8 ± 17.0
DBP (mmHg)	69.1 ± 9.1	73.5 ± 9.0
Duration of diabetes (year)		14.7 ± 11.5
HbA1c (%)		6.7 ± 0.6
Creatinine (mg/dl)		0.87 ± 0.26
eGFR (mL/min/1.73 m^2^)		63.5 ± 14.8
Neuropathy, n (%)		8 (26.7)
Retinopathy, n (%)		3 (10.0)
Nephropathy, n (%)		6 (20.0)
Anti-diabetes treatment		
Diet only, n (%)		1 (3.0)
SU, n (%)		0 (0)
Glinide, n (%)		2 (6.7)
DPP-4 inhibitor, n (%)		21 (70.0)
Biguanide, n (%)		15 (50.0)
Thiazolidine, n (%)		5 (16.7)
SGLT-2 inhibitor, n (%)		7 (23.3)
α-glucose inhibitor, n (%)		7 (23.3)
GLP-1 receptor, n (%)		3 (10.0)
Insulin, n (%)		6 (20.0)

Sex, by χ^2^ test. Age, by Welch test. *BMI* Body mass index, *SBP* Systolic blood pressure, *DBP* Diastolic blood pressure, *eGFR* estimated glomerular filtration rate, *SU* sulfonylureas, *DPP-4 inhibitor* dipeptidyl peptidase-4 inhibitor, *SGLT-2 inhibitor* sodium-glucose transporter-2 inhibitor, *GLP-1 receptor* Glucagon-like peptide-1 receptor

### Comparison of the diabetic and control groups

The FGM data of the diabetic group and control group are shown in Additional file 2: Table S1. The average IQR (AIQR) was 17.3 ± 4.3 mg/dl for the control group, and significantly higher for the diabetic group (30.1 ± 11.7 mg/dl, p < 0.001). The TBR/TIR/TAR were 2.7 ± 5.3/96.1 ± 5.5/1.3 ± 2.1% in the control group and 1.6 ± 2.4/85.8 ± 12.8/12.6 ± 12.5% in the diabetes group. The TIR was significantly lower (p < 0.001), while TAR was significantly higher (p < 0.001) in the diabetic group. There were no notable differences between the two groups with respect to the minimum, TBR, and LBGI.

### Correlation between AIQR and CGM index in the diabetic group

**Fig. 1:**
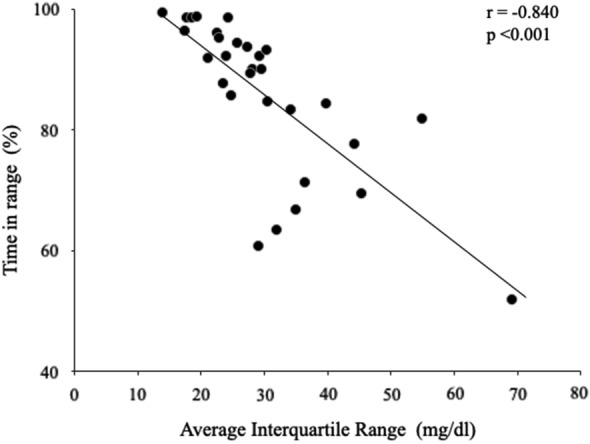
Correlation between average IQR and TIR in patients of the diabetic group. Average IQR correlated negatively with TIR (r = − 0.840, p < 0.001). IQR (interquartile range): TIR (time-in-range) (70–180 mg/dl)

Table 2 summarizes the results of analysis of the correlation between AIQR and CGM metrics in the diabetic group. The AIQR correlated negatively (r = − 0.840, p < 0.001, Fig. 1) with TIR, and positively with maximum, SD, CV, MODD, HGBI, and TAR (p < 0.001, each). However, there was no correlation between AIQR and the hypoglycemia indexes of minimum, LBGI, and TBR. Analysis of the data of all 20 patients with hypoglycemia showed a significant correlation between AIQR and TBR (p = 0.032, r = 0.481).

**Table 2:** Correlation between average IQR and CGM index in the diabetic patients

	Correlation coefficient	p value
TIR	−0.840	< 0.001
AG	0.805	< 0.001
Maximum	0.839	< 0.001
Minimum	0.181	0.339
SD	0.822	< 0.001
CV	0.512	0.004
MODD	0.949	< 0.001
HBGI	0.887	< 0.001
LBGI	0.038	0.844
TAR	0.868	< 0.001
TBR	0.007	0.970

P values by Spearman’s rank correlation coefficient

*IQR* interquartile range, *TIR* Time in range (70–180 mg/dl), *AG* average interstitial glucose, *SD* standard deviation, *CV* coefficient of variation, *MODD* mean of daily difference of blood glucose, *HBGI* high blood glucose index, *LBGI* low blood glucose index, *TAR* Time above range (> 180 mg/dl), *TBR* Time below range (< 70 mg/dl)

### Comparison of AIQR according to TIR achievement in the diabetic group

We compared the AIQR of the diabetic group based on the achievement of TIR > 70% and TIR > 90% (Table 3). The analysis showed 25 patients achieved TIR > 70% and 5 patients that did not, while 14 patients achieved TIR > 90% and 16 did not. The AIQR of the group that achieved TIR > 70% was significantly lower (27.6 ± 9.1 mg/dl) than that of the group that did not (42.2 ± 16.3 mg/dl, p = 0.017). Similarly, the AIQR of the group that achieved TIR > 90% was significantly lower (22.6 ± 4.8 mg/dl) than that of the group that did not (36.7 ± 12.6 mg/dl, p < 0.001).

**Table 3:** Comparison of average IQR according to TIR achievement in diabetic patients

Average IQR by achieving TIR > 70%			
	Achievement group (n = 25)	non-Achievement group (n = 5)	p value
Average IQR (mg/dl)	27.6 ± 9.1	42.2 ± 16.3	0.017

Average IQR by achieving TIR > 90%			
	Achievement group (n−14)	non-Achievement group (n = 16)	p value
Average IQR (mg/dl)	22.6 ± 4.8	36.7 ± 12.6	< 0.001

Data are mean ± SD

P values by Mann–Whitney test

*IQR* interquartile range, *TIR* time in range (70–180 mg/dl)

To evaluate the AIQR cutoff values for the groups that achieved TIR > 70% and TIR > 90%, ROC curves were generated and the areas under the curves (AUC) were computed. ROC curve analysis showed an AIQR cutoff value of 28.3 mg/dl (AUC = 0.848, 95% CI 0.621–0.950) for the group that achieved TIR > 70%, and 22.9 mg/dl (AUC = 0.929, 95% CI 0.778–0.980) for the group that achieved TIR > 90% (Additional file 3: Figure S2).

**Fig. 2:**
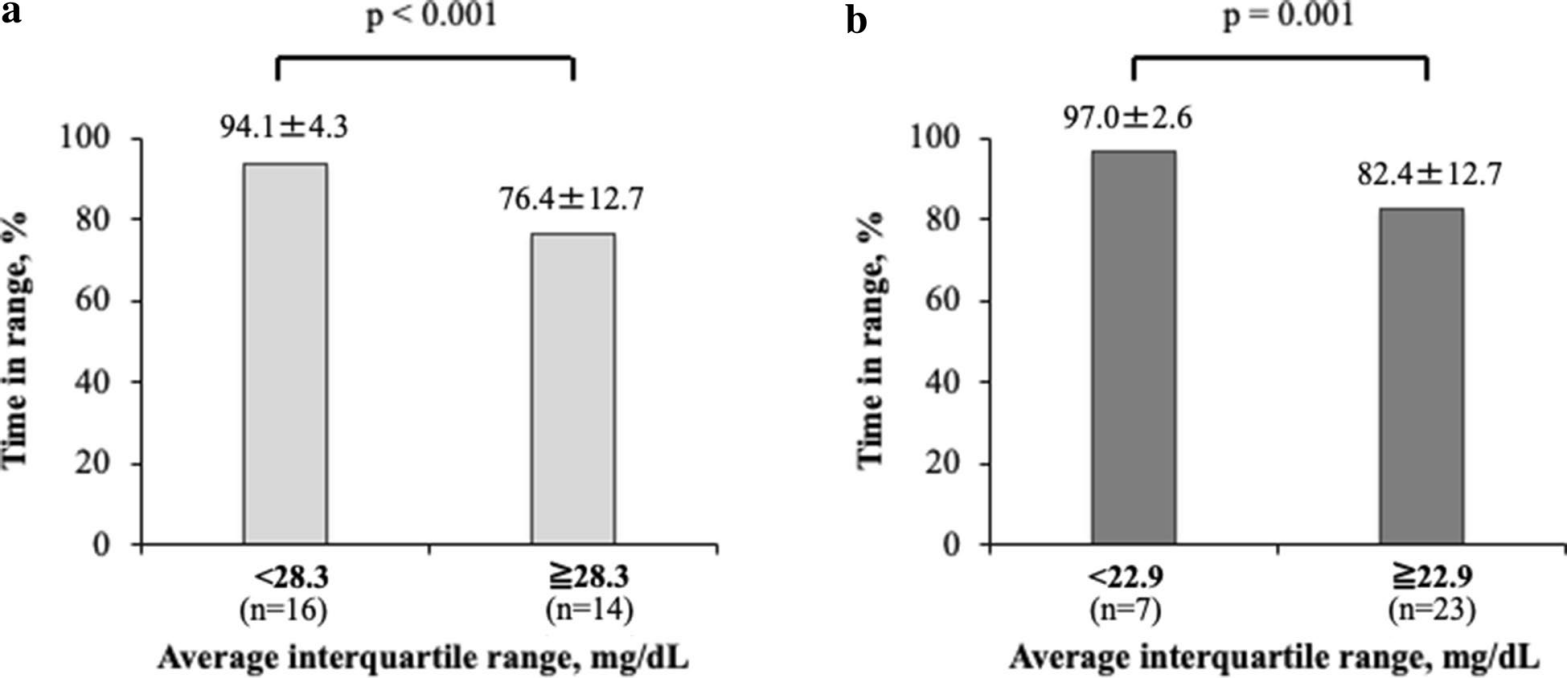
Comparison of TIR by average IQR. **a** Note the significant difference in TIR when AIQR cutoff was 28.3 mg/dl (p < 0.001). **b** Note the significant difference in TIR when the AIQR cutoff was 22.9 mg/dl (p = 0.001). Data are mean ± standard deviation. IQR (interquartile range): TIR (time-in-range) (70–180 mg/dl)

Next, we used an AIQR of 28.3 mg/dl as the cutoff value and compared the data with TIR. The TIR was 94.1 ± 4.3% in the AIQR < 28.3 mg/dl group and 76.4 ± 12.7% (p < 0.001) in the AIQR ≥ 28.3 mg/dl group. On the other hand, using an AIQR of 22.9 mg/dl as the cutoff value, the TIR were 97.0 ± 2.6% and 82.4 ± 12.7% (p = 0.001) for the AIQR < 22.9 and ≥ 22.9 mg/dl groups, respectively (Fig. 2).

### Comparison of the hypoglycemia and non-hypoglycemia subgroups

Table 4 shows the results of comparison of CGM metrics in the presence of hypoglycemia. Hypoglycemia was noted in 20 of the 30 patients of the diabetic group, and the hypoglycemia indexes of TBR and LGBI were significantly higher in the hypoglycemia group than the non-hypoglycemia group (p < 0.001, each). However, there was no notable difference in AIQR in the presence of hypoglycemia. AG, GMI, and TAR were all significantly higher in the non-hypoglycemia group (p = 0.005, p = 0.005, p = 0.037).

**Table 4:** Hypoglycemia versus non-hypoglycemia in diabetic patients

	Mean ± SD		p value
	Hypoglycemia (n = 20)	non-Hypoglycemia (n = 10)	
AG (mg/dl)	123.8 ± 18.6	145.9 ± 19.2	0.005^a^
Maximum (mg/dl)	208.9 ± 34.0	233.6 ± 39.3	0.095
Minimum (mg/dl)	73.3 ± 7.9	96.1 ± 13.6	< 0.001
SD (mg/dl)	33.5 ± 9.1	35.1 ± 9.2	0.649^a^
CV (mg/dl)	26.9 ± 4.5	24.0 ± 5.1	0.127^a^
GMI (%)	24.0 ± 3.1	27.7 ± 3.2	0.005^a^
TAR (%)	9.9 ± 11.5	18.0 ± 13.1	0.037
TIR (%)	87.7 ± 12.5	82.0 ± 13.1	0.179
TBR (%)	2.3 ± 2.6	0	< 0.001
LBGI (%)	2.5 ± 1.9	0.7 ± 0.5	< 0.001
MODD (mg/dl)	28.5 ± 12.5	31.1 ± 9.2	0.271
AIQR (mg/dl)	29.7 ± 13.7	30.8 ± 6.5	0.271

Data are mean ± SD

Unmarked *p* values, by Mann–Whitney test

*AG* average interstitial glucose, *SD* standard deviation, *CV* coefficient of variation, *GMI* Glucose Management Indicator, *TAR* time above range (> 180 mg/dl), *TIR* time in range (70–180 mg/dl), *TBR* time below range (< 70 mg/dl), LBGI, low blood glucose index, *MODD* mean of daily difference of blood glucose, *AIQR* average interquartile range

^a^By Student’s t test when variances were equal as determined by an F test, otherwise with Welch’s test

## Discussion

The major findings of the present study using the FGM were the following: (1) AIQR correlated negatively with TIR, (2) the higher the TIR lower the variability seen by the AGP, (3) AIQR correlated positively with SD, CV, and MODD, and (4) Lack of relationship between AIQR and hypoglycemia.

Our study also established the AIQR cutoff values for achieving the TIR target values. AGP is an excellent tool that clearly and visually presents the trends in interstitial glucose variability for the individual patient [9]; our study showed that AIQR is a valuable index for use in AGP.

Our data showed that AIQR correlates negatively with TIR, and that the longer the time interstitial glucose is maintained within the range of 70 to 180 mg/dl, the smaller the variation range in AGP. TIR refers to the percent time during the 24-h period when blood glucose is within the range of 70–180 mg/dl. TIR provides a more complete picture of blood glucose than HbA1c, and hence makes it possible to offer personalized treatment options [10]. Similar to HbA1c, TIR is reported to correlate strongly with the risk of retinopathy and/or onset of microalbuminuria [2]. Other studies found that TIR is independent of HbA1c and that it is associated with cardiovascular autonomic neuropathy [11]. Furthermore, TIR correlates strongly with HbA1c [12], and was recently recommended by the American Diabetes Association (ADA) for use along with HbA1c for targeted blood glucose control.

IQR is in the 25–75th percentile range on the AGP, and is an excellent visual indicator of interstitial glucose variability [13, 14]; although there is no reported relationship between IQR and standardized CGM metrics. To our knowledge, our study is the first to define the AIQR-TIR relationship. Importantly, setting the cutoff values for AIQR makes it easier to evaluate treatment goals especially achieving the TIR targets. Following ADA’s target of TIR > 70% in T2DM patients, we established the AIQR cutoff values of 28.3 mg/dl for TIR > 70% and 22.9 mg/dl for a stricter adherence to TIR > 90%. These indexes are useful markers to use when setting target treatment values using the AGP. These parameters are useful as visual indicator to highlight the therapeutic target with the patients.

The IQR has also been discussed as a marker of interday variations [15]. Another strong aspect of our study was the strong correlation between AIQR and the SD and CV (which are markers of intraday variations), in addition to the correlation between AIQR and MODD (which is a marker of interday variation). In this regard, Monnier et al. [16] proposed the use of indexes that can evaluate blood glucose variability in addition to chronic hyperglycemia and hypoglycemia, as a strategy for monitoring blood glucose to inhibit progression of cardiovascular disorders. The results of the present study suggest that IQR is useful index for evaluation of intra- and interday variations in interstitial glucose levels. In practical clinical settings from hereon, a large IQR will require analysis of the factors behind interday variations, as well as necessitate evaluation of the factors responsible for intraday variations, including, for example, postprandial glucose and low blood glucose levels.

Our analysis showed the lack of any association between AIQR and presence of hypoglycemia, and that low interstitial glucose is associated with AG and LBGI. These results suggest that AIQR is not suitable for use to predict the extent of hypoglycemia; and thus, we advise instead to visually evaluate each case when assessing low interstitial glucose in AGP. It is noteworthy that a number of investigators found close associations between low blood glucose and certain markers, such as AG and LBGI [17, 18], similar to our findings. Therefore, AG, CV, LBGI and other CGM metrics can also be used with other indexes to evaluate objectively the risk of hypoglycemia in clinical settings.

There were following limitations in this study. The FGMS^®^ System FreeStyle Libre Pro System can measure up to 14 consecutive days. However, recent studies have reported that measurements taken on day 1 and those after day 12 are inaccurate [19]. Based on this limitation of the system, we decided to use the data of only day 2 to day 8 (7 days), excluding the data of day 1, in our analysis. Since The International Consensus Statement recommends AGP analysis of the data of 14 days [20], further studies are necessary to verify whether the results of 7-day analysis are identical to those of 14 days. In addition, we did not record interstitial glucose density of ≥ 500 mg/dl based on the use of FGM, and accordingly, or study did not include patients with poor blood glucose control. This is an important limitation to mention here because the results for such patients may be different from those obtained in the present study. And furthermore, this research work was a cross-sectional study conducted at two facilities with only a relatively small number of patients. Therefore, it was not possible to analyze this subject according to hypoglycemia or medication type. To substantiate the results of this study, a multi-center study of a large number of cases needs to be performed.

## Conclusion

This study noted, for the first time, a strong correlation between TIR and interstitial glucose variability range that can be understood visually using the AGP. The study also provided the AIQR cutoff values for achieving TIR target values. Furthermore, the interstitial glucose variability range in AGP was strongly associated with indexes of intra- and interday variations; though there was no association with LBGI. Our results may provide new perspectives in the yet-to-be established methods for evaluating AGP in practical clinical settings.

## Supplementary information

**Additional file 1: Figure S1.** Ambulatory Glucose Profile (AGP). The dark blue line in the center represents the median value. The two outer lines represent the 25th percentile at the bottom and the 75th percentile at the top, and the black arrow width represents the inter-quartile range (IQR).

**Additional file 2: Table S1.** Results of Flash glucose monitoring for patients with type 2 diabetes and the control non-diabetic subjects.

**Additional file 3: Figure S2.** ROC curve by TIR achievement using data of patients of the diabetic group. ROC curve analysis demonstrated an IQR cutoff of 28.3 mg/dl (area under the curve = 0.848, 95% CI 0.621–0.950) for achieving TIR > 70%, and 22.9 mg/dl (area under the curve = 0.929, 95% CI 0.778–0.980) for achieving TIR > 90%. IQR (interquartile range): TIR (time-in-range) (70–180 mg/dl).

## Data Availability

The datasets used and/or analysed during the current study are available from the corresponding author on reasonable request.

## Abbreviations

CGM: Continuous glucose monitoring
AGP: Ambulatory glucose profile
FGM: Flash glucose monitoring
IQR: Inter-quartile range
T2DM: Type 2 diabetes mellitus
average IQR: Average inter-quartile range
TIR: Time in range
SD: Standard deviation
CV: Coefficient of variation
MODD: Mean of daily difference of blood glucose
ROC analysis: Receiver Operating Characteristic analysis
IDR: Inter-decile range
AG: Average interstitial glucose level
TAR: Time above range
TBR: Time below range
GMI: Glucose management indicator
LBGI: Low blood glucose index
HBGI: High blood glucose index
HbA1c: Hemoglobin A1c
eGFR: Estimated glomerular filtration rate
AUC: Area under the curve
BMI: Body mass index
DPP-4 inhibitor: Dipeptidyl peptidase-4 inhibitor
95%CI: 95% confidence interval
