# Supplementary figures and images for: Relationship between interstitial glucose variability in ambulatory glucose profile and standardized continuous glucose monitoring metrics; a pilot study
Source: Diabetol Metab Syndr. 2020 Aug 12;12:70. doi: 10.1186/s13098-020-00577-5 (PMC7424649; doi:10.1186/s13098-020-00577-5)

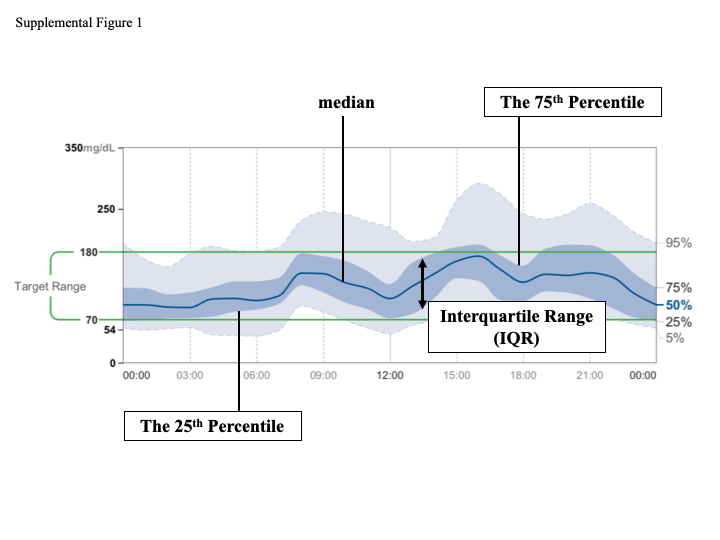

Supplement: Supplementary file 1 — Additional file 1: Figure S1. Ambulatory Glucose Profile (AGP). The dark blue line in the center represents the median value. The two outer lines represent the 25th percentile at the bottom and the 75th percentile at the top, and the black arrow width represents the inter-quartile range (IQR). [file 13098_2020_577_MOESM1_ESM.tiff]

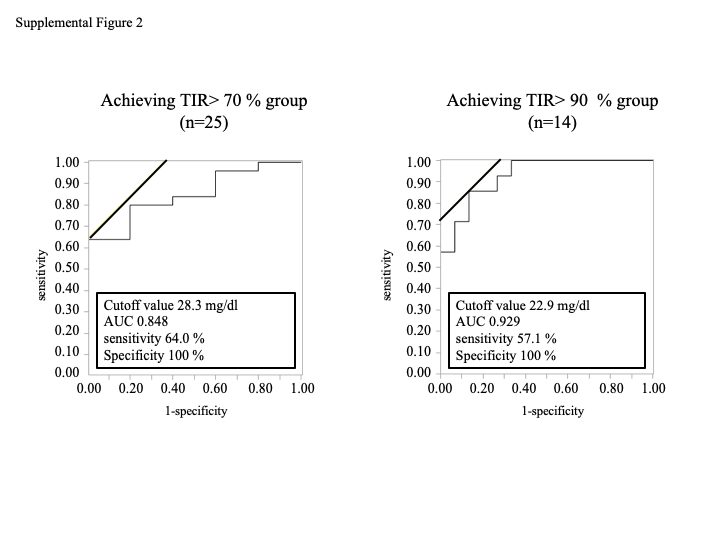

Supplement: Supplementary file 3 — Additional file 3: Figure S2. ROC curve by TIR achievement using data of patients of the diabetic group. ROC curve analysis demonstrated an IQR cutoff of 28.3 mg/dl (area under the curve = 0.848, 95% CI 0.621–0.950) for achieving TIR > 70%, and 22.9 mg/dl (area under the curve = 0.929, 95% CI 0.778–0.980) for achieving TIR > 90%. IQR (interquartile range): TIR (time-in-range) (70–180 mg/dl). [file 13098_2020_577_MOESM3_ESM.tiff]
