# Supplementary material for: Relationship between interstitial glucose variability in ambulatory glucose profile and standardized continuous glucose monitoring metrics; a pilot study
Source: Diabetol Metab Syndr. 2020 Aug 12;12:70. doi: 10.1186/s13098-020-00577-5 (PMC7424649; doi:10.1186/s13098-020-00577-5)
Supplement: Supplementary file 2 — Additional file 2: Table S1. Results of Flash glucose monitoring for patients with type 2 diabetes and the control non-diabetic subjects. [file 13098_2020_577_MOESM2_ESM.doc]

**Table S1.** Results of Flash glucose monitoring for patients with type 2 diabetes and the control non-diabetic subjects.

|  | Control group | Diabetes group | p value |
| --- | --- | --- | --- |
| Median (mg/dL) | 103.5±10.5 | 130.9±22.3 | <0.001^a^ |
| AIQR (mg/dL) | 17.3±4.3 | 30.1±11.7 | <0.001 |
| Mean (mg/dL) | 105.2±10.5 | 131.2±21.3 | <0.001^a^ |
| Maximum (mg/dL) | 168.5±22.2 | 217.1±37.1 | <0.001 |
| Minimum (mg/dL) | 74.7±9.5 | 80.9±14.8 | 0.332 |
| GMI (%) | 5.8±0.3 | 6.4±0.5 | <0.001 |
| SD (mg/dL) | 20.7±5.3 | 34.1±9.0 | <0.001^a^ |
| CV (mg/dL) | 19.8±4.5 | 25.9±4.8 | <0.001^a^ |
| TBR (%) | 2.7±5.3 | 1.6±2.4 | 0.234 |
| TIR (%) | 96.1±5.5 | 85.8±12.8 | <0.001 |
| TAR (%) | 1.3±2.1 | 12.6±12.5 | <0.001 |
| MODD (mg/dL) | 17.5±4.1 | 29.4±11.4 | <0.001 |
| LBGI - | 2.0±1.3 | 1.9±1.8 | 0.518 |
| HBGI - | 1.5±0.9 | 4.6±2.5 | <0.001 |

Data are mean ± SD. Measured by the Flash glucose monitoring system. The FGM data were collected over seven consecutive days, and the values ​​in the table represent the mean of seven days. P values are for differences between the two groups.

^a^By the Student’s t test when the variance was equal as determined by an F test, otherwise with Welch’s test. Unmarked *p* values, by Mann-Whitney test.

AIQR, average interquartile range: GMI, Glucose Management Indicator; a new term for estimating HbA1c from continuous glucose monitoring: SD, standard deviation: CV, coefficient of variation: TBR, Time below range (<70 mg/dl): TIR, Time in range (70-180 mg/dL): TAR, Time above range (>180 mg/dL): MODD, mean of daily difference of blood glucose: LBGI, low blood glucose index: HBGI, high blood glucose index.
